# 1-Ethyl-3-methylimidazolium acetate as a highly efficient organocatalyst for cyanosilylation of carbonyl compounds with trimethylsilyl cyanide

**DOI:** 10.1038/srep42699

**Published:** 2017-02-15

**Authors:** Bakhtar Ullah, Jingwen Chen, Zhiguo Zhang, Huabin Xing, Qiwei Yang, Zongbi Bao, Qilong Ren

**Affiliations:** 1 Key Laboratory of Biomass Chemical Engineering of Ministry of Education, College of Chemical and Biological Engineering, Zhejiang University, Hangzhou 310027, China

## Abstract

1-Ethyl-3-methylimidazolium acetate is introduced as a robust organocatalyst for solvent-free cyanosilylation of carbonyl compounds with trimethylsilyl cyanide (TMSCN). The catalyst loading can be reduced to as low as 0.1–0.0001 mol % under mild reaction conditions, giving considerably high TOF values from 10,843 h^−1^ to 10,602,410 h^−1^ in the field of organocatalyzed transformations. The present protocol not only tolerates with extensive carbonyl compounds but also provides somewhat insight into the mechanism of ionic liquids (ILs)-catalyzed reactions.

Cyanohydrins^1^,^2^,^3^,^4^ are one of the key synthetic intermediates since they can be elaborated into a number of valuable organic compounds, i.e. β-amino alcohols, α-hydroxy acids, α-amino acids, etc.^5^,^6^ Hence, much effort and consideration have been devoted to the synthesis of cyanohydrins^1^,^2^,^3^,^4^. Lapworth and Arthur were among the first to report on the synthesis of cyanohydrins by the addition of hydrogen cyanide (HCN) to carbonyl compounds^7^. However, due to the toxicity and difficulties in handling of HCN, a number of alternative cyanating reagents with less harmful and easily manageable properties have been subsequently introduced^1^,^2^,^3^,^4^. Among other cyanating reagents, TMSCN is one of the most accessed reagents for cyanohydrins synthesis, allowing them to be prepared as cyanohydrin trimethylsilyl ethers^1^,^2^,^3^,^4^,^7^,^8^,^9^,^10^,^11^,^12^,^13^,^14^. In this respect, the development of efficient catalysts for the addition of TMSCN to carbonyl compounds has been the focal research point. As a consequence, various Lewis acids, Lewis bases, metal alkoxides, as well as inorganic salts have been successfully employed in promoting this transformation^3^,^12^,^13^,^14^,^15^,^16^,^17^,^18^,^19^,^20^,^21^,^22^,^23^,^24^.

In the past decades, organocatalysis has received much attention and started to serve as the practical method in synthetic paradigm^25^,^26^,^27^,^28^,^29^. The operational simplicity and readily availability of mostly inexpensive bench-stable catalysts compelled organocatalysis to develop into an important segment in common with metal- and bio-catalysis^25^,^30^. Although organic species such as amines, phosphines, *N*-heterocyclic carbenes (NHCs) etc. have been employed as catalysts for the cyanosilylation of carbonyl compounds with TMSCN^19^,^22^,^25^,^26^,^31^,^32^,^33^,^34^,^35^, most of them are encountered with some impediments, especially high catalyst loading and long reaction time. Therefore, some practical and highly efficient organocatalysts are still extremely desirable.

ILs have wide applications in chemistry such as powerful solvents, electrolytes, gas handling media, etc. due to their inherent assets, i.e. low vapor pressure, non-flammability, thermal stability, conductivity, and high gas solubility^36^,^37^,^38^,^39^,^40^,^41^. Beyond the applications mentioned above, imidazolium-based ILs have also been demonstrated to be efficient organocatalysts for the degradation of cellulose^42^,^43^. Loh and co-workers reported that [OMIM][PF_6_] is an efficient and recyclable reaction media for the cyanosilylation of aldehydes^44^. Later on, Lee and co-workers demonstrated that the catalytic activity of scandium triflate in [BMIM][SbF_6_] was dramatically improved for the cyanosilylation of various aldehydes and ketones^45^. In addition, polymer supported ILs have also been developed as catalysts for this transformation but with low catalytic efficiency^41^,^46^,^47^,^48^. In continuation of our efforts to develop practically useful organocatalysts for the cyanosilylations^13^,^28^, we envisaged that the ionic networks (organic cations and anions) in ILs may act as a stable and robust catalytic species for cyanosilylations^41^,^42^,^43^,^44^,^45^,^46^,^47^,^48^. Herein, we present that 1-ethyl-3-methylimidazolium acetate ([EMIM]OAc) is a highly efficient catalyst for such a transformation.

## Results and Discussion

We started our studies by examination of the catalytic activity of a set of imidazolium-based ILs, using the cyanosilylation of benzaldehyde with TMSCN as a model reaction (Table 1). In the presence of 0.01 mol % of catalyst loading, imidazolium-based ILs with various counter anions demonstrated different catalytic activities. Imidazolium-based ILs with counter anions such as OAc^−^, SCN^−^ and (EtO)_2_PO_2_^−^ were obviously efficient even with lower catalyst loading (0.005 mol %) in promoting this transformation, giving the product in excellent yield within 5 minutes (Table 1, entries 1–3). Imidazolium-based ILs with counter anions such as Cl^−^, N(CN)_2_^−^, ClO_4_^−^, EtOSO_3_^−^, BF_4_^−^ and Br^−^ generally gave the product in low to moderate yields (Table 1, entries 4–9)^49^. Among all the imidazolium-based ILs tested, [EMIM]OAc was the most efficient catalyst, which is in accordance with its basicity and accordingly underlines the possible anion-activation mechanistic mode. Furthermore, we performed several control experiments by switching imidazolium cation to other types of cations i.e., tetrabutylphosphonium, ammonium, sodium as well as potassium etc. (Table 1, entries 10–13), most of them were not efficient for this reaction with an exception of tetrabutylphosphonium cation (78% yield), suggesting that both cations and anions were essential for catalytic efficiencies. It is worth of noting that the catalytic activities of ILs and other salts having acetate anions seemed to agree with the interaction strength between cation and anion pairs^49^. In the absence of catalyst, only trace amount of the desired product was detected (Table 1, entry 14). Among all the screened catalytic protocols, [EMIM]OAc (**1a**) was found as an efficient catalyst for cyanosilylation of benzaldehyde with TMSCN. In view of the high efficacy and readily availability of **1a**, we employed it as the corresponding catalyst for further cyanosilylation optimizations.

In the next, we optimized the reaction conditions in terms of solvents and catalyst loadings, and the results were summarized in Table 2. In the presence of 0.01 mol % of **1a**, the reaction performed in toluene, CH_2_Cl_2_, CHCl_3_, and THF proceeds smoothly to give the product with a yield of 94%, 83%, 88%, and 85%, respectively (Table 2, entries 1–4). The reaction in CH_3_CN was substantially suppressed due to its high polarity, only 48% yield was obtained in 5 minutes (Table 2, entry 5). To our delight, the reaction under neat conditions affords the product in quantitative yield (Table 2, entry 6). Also with reduced amount of catalyst, i.e., 0.005 mol % of **1a**, we obtained 94% yield within 5 minutes under solvent-free conditions (Table 2, entry 7). We were pleased to find that the catalyst loading of **1a** can further be reduced to 0.001 mol % with 87% yield at a prolonged reaction time (Table 2, entry 8). Using acetophenone as a substrate, the catalyst loading of **1a** needs to increase up to 0.1 mol % for completing the reaction within 5 minutes (Table 2, entry 10). We selected 0.005 mol % and 0.1 mol % catalyst loading of **1a** with neat reaction conditions as optimal choices for further applications in cyanosilylation of aldehydes and ketones, respectively.

Under the optimized reaction conditions, various aldehydes were subjected to this protocol and the results were summarized in Table 3. The cyanosilylation of benzaldehyde with TMSCN produced 94% yield within 5 minutes in the presence of 0.005 mol % of **1a** under neat reaction conditions (Table 3, entry 1). Benzaldehyde derivatives substituted with electron withdrawing groups (EWGs) reacted much faster than those having electron donating groups (EDGs). Even with as low as 0.001 mol % of **1a**, benzaldehyde derivatives having electron withdrawing substituents such as *p*-chloro and *m*-fluoro afforded excellent yield (Table 3, entries 2–3). In contrast, benzaldehyde derivatives substituted with EDGs such as 3-methoxyl group, a satisfactory yield was obtained when the catalyst loading was increased to 0.1 mol % (Table 3, entry 4). Cinnamaldehyde required prolonged reaction time to get a satisfactory yield, and was essentially converted to 1,2–adducts, meanwhile leaving the olefin functional group unchanged (Table 3, entry 5)^12^,^50^. In line with the electron poor aromatic aldehydes, 0.001 mol % of **1a** was also effective enough for aliphatic aldehydes (Table 3, entries 7–9). To our delight, the catalyst loading of **1a** can further be reduced to 0.0001 mol % for aliphatic aldehydes with a remarkable TOF value 10,602,410 h^−1^ (Table 3, entry 8).

With the optimized reaction conditions in hand, ketones were employed as substrates and the results were summarized in Table 4. In the presence of 0.1 mol % of **1a**, both aromatic and aliphatic ketones provided excellent yield (Table 4, entries 1–8). The substituent on aromatic ketones did not show considerable influence on the reaction outcomes. Under the same reaction conditions, **1a** is also capable to convert the relatively unreactive benzophenone into its product with excellent yield (Table 4, entry 9)^32^.

**Figure 1:**
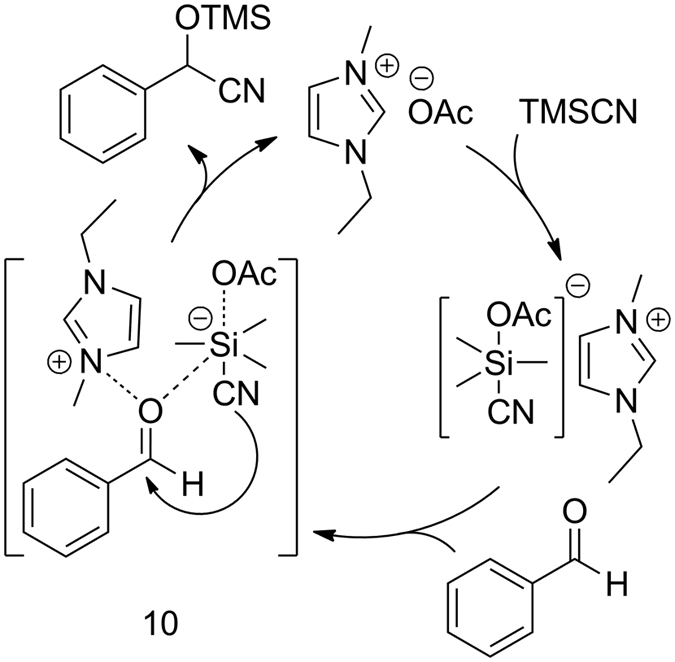
Proposed mechanism for the cyanosilylation of carbonyl compounds (benzaldehyde as a model substrate)^a^. ^a^Synergistic mode of activation by acetate anion (OAc^−^) and imidazolium cation of [EMIM]OAc (**1a**).

The exact mechanism for **1a** catalyzed cyanosilylation is still unclear. Our original mechanistic proposal was based on a synergistic activation mode, i.e., imidazolium cation interacts with carbonyl compounds by facilitating the attack of acetate anion activated TMSCN and it was considered that the reaction may proceed through hypervalent silicate intermediate 10, (Fig. 1)^29^,^51^,^52^,^53^,^54^. The formation of hypervalent silicate intermediate between acetate anion and TMSCN was confirmed by ^1^H NMR spectra of TMSCN and a 1:1 mixture of TMSCN and **1a** in CDCl_3_ (for detail of ^1^H NMR spectra, see Supplementary page S13). On the other hand, a connection with recently discovered “carbene from imidazolium-based ILs” was also under consideration^55^,^56^,^57^,^58^,^59^,^60^,^61^, in which the *in situ* generated NHC by deprotonation of carbon-2 at imidazolium cation with its acetate anion may act as an efficient catalyst for cyanosilylation of carbonyl compounds^3^,^26^,^31^,^32^,^33^,^34^,^35^,^59^. In order to gain insight into this mechanistic mode, we intentionally blocked the C-2 position of imidazolium cation with a methyl group by preparing 1-ethyl-2,3-dimethylimidazolium acetate [EMMIM]OAc and employed in the cyanosilylation of benzaldehyde^62^. Under relatively identical reaction conditions, cyanosilylation of benzaldehyde using [EMMIM]OAc as a catalyst gave 88% yield, which was comparable to the 94% yield afforded by **1a**. As a consequence, we postulated that the *in situ* generated NHC may not play a significant role in the catalytic performance of **1a** and a synergistic activation mode is probably the main reaction pathway (Fig. 1).

## Conclusions

In conclusion, we have developed a highly efficient cyanosilylation reaction of carbonyl compounds using commercially and readily available [EMIM]OAc (**1a**) as an organocatalyst. In the presence of 0.0001–0.1 mol % of [EMIM]OAc, various aldehydes and ketones were converted to their corresponding products in excellent yields. The catalyst is truly active giving quite high TOF values from 10,843 h^−1^ to 10,602,410 h^−1^, which is among the most efficient organocatalysts. Mechanistic investigations based on experimental results revealed that the reaction operates via a synergistic activation mode, namely, imidazolium cation interacts with carbonyl compounds by facilitating the attack of acetate anion activated TMSCN. From a practical point of view, this protocol offers a cost effective and facile way for the synthesis of cyanohydrins. Asymmetric cyanosilylation of carbonyl compounds using imidazolium-based chiral ILs is under investigation in our laboratory and will be reported in due course.

## Methods

### General procedure for cyanosilylation of carbonyl compounds (benzaldehyde as a typical example with TMSCN catalyzed by IL [EMIM]OAc (1a))

#### Caution

TMSCN must be used in a well-ventilated hood due to its high toxicity.

The reaction was carried out by placing freshly distilled TMSCN (1.2 mmol), **1a** (0.005 mol %), and a teflon-coated magnetic stir bar into a Pyrex-glass screw cap vial. The solvent-free reaction was initiated by addition of freshly distilled benzaldehyde (1.0 mmol) and was stirred vigorously at room temperature. The reaction was monitored by TLC. After 5 minutes, the yield of benzaldehyde to its corresponding silylated cyanohydrin was determined by ^1^H NMR as 94%. For all other carbonyl compounds the same procedure with the same amount of reagents were used, as described earlier in Tables 1, 2, 3, 4. In case of aldehydes the yields were determined by ^1^H NMR, whereas the yields of ketones were isolated by flash column chromatography on silica gel (eluent: n–hexane/ethyl acetate 40:1). All silylated cyanohydrin products of respective carbonyl compounds with TMSCN were confirmed by comparison of their ^1^H NMR spectral data with those of authentic data^13^.

## Additional Information

**How to cite this article**: Ullah, B. *et al*. 1-Ethyl-3-methylimidazolium acetate as a highly efficient organocatalyst for cyanosilylation of carbonyl compounds with trimethylsilyl cyanide. *Sci. Rep.* **7**, 42699; doi: 10.1038/srep42699 (2017).

**Publisher's note:** Springer Nature remains neutral with regard to jurisdictional claims in published maps and institutional affiliations.

## Supplementary Material

Supplementary Information

## Figures and Tables

**Table 1:**
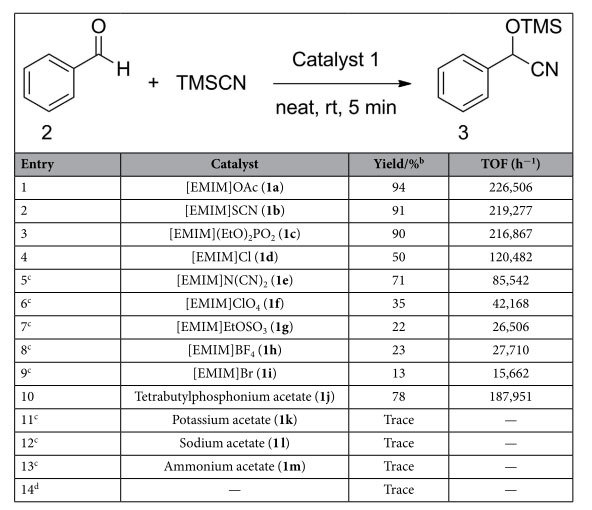
Survey of imidazolium-based ionic liquids as catalysts in the cyanosilylation of benzaldehyde with TMSCN^a^.

^a^The reaction was carried out with benzaldehyde (1.0 mmol) and TMSCN (1.2 mmol) in the presence of 0.005 mol % of catalysts for 5 minutes under neat conditions. ^b^Yield was determined by ^1^H NMR. ^c^0.01 mol % of catalyst was used. ^d^Without catalysts.

**Table 2:**
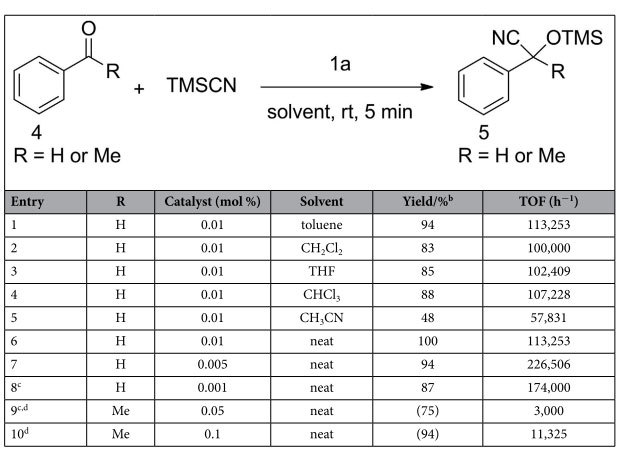
Optimization of [EMIM]OAc (1a) catalyzed cyanosilylations^a^.

^a^The reaction was carried out with benzaldehyde/acetophenone (1.0 mmol) and TMSCN (1.2 mmol) in the presence of 0.001–0.1 mol % of **1a** for 5 minutes. ^b^Determined by ^1^H NMR (isolated yields are given in parenthesis). ^c^30 minutes of reaction time. ^d^TON values for acetophenone are 1500 and 940 for entries 9 and 10, respectively.

**Table 3:**
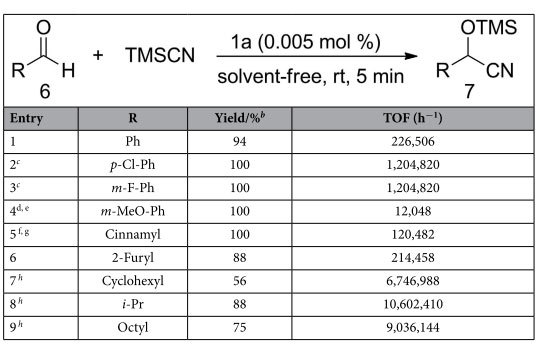
Substrates scope of [EMIM]OAc (1a) catalyzed cyanosilylation of aldehydes^a^.

^a^The reaction was carried out with various aldehydes (1.0 mmol) and TMSCN (1.2 mmol) in the presence of 0.001–0.1 mol % of **1a** for 5 minutes under neat conditions at room temperature. ^b^Determined by ^1^H NMR. ^c^0.001 mol % of **1a**. ^d^0.1 mol % of **1a**. ^e^The yield is 40% with 0.01 mol % of **1a** in 30 minutes. ^f^0.01 mol % of **1a**. ^g^The yield is 88% with 0.005 mol % of **1a** in 30 minutes. ^h^0.0001 mol % of **1a**, TON values for aliphatic aldehydes (entries 7–9) are 560,000, 880,000, and 750,000, respectively.

**Table 4:**
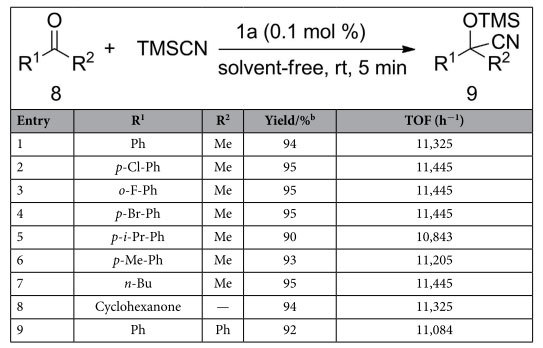
Substrates scope of [EMIM]OAc (1a) catalyzed cyanosilylation of ketones^a^.

^a^The reaction was carried out with various ketones (1.0 mmol) and TMSCN (1.2 mmol) in the presence of 0.1 mol % of **1a** for 5 minutes under neat conditions at room temperature. ^b^Isolated yields.

## References

[b1] Gregory R. J. Cyanohydrins in nature and the laboratory: biology, preparations, and synthetic applications. Chem Rev 99, 3649–3682 (1999). 11849033 10.1021/cr9902906

[b2] Brunel J. M. & Holmes I. P. Chemically catalyzed asymmetric cyanohydrin syntheses. Angew Chem Int Ed 43, 2752–2778 (2004). 10.1002/anie.200300604 15150747

[b3] North M., Usanov D. L. & Young C. Lewis acid catalyzed asymmetric cyanohydrin synthesis. Chem Rev 108, 5146–5226 (2008). 19067648 10.1021/cr800255k

[b4] Pellissier H. Enantioselective titanium-catalyzed cyanation reactions of carbonyl compounds. Adv Synth Catal 357, 857–882 (2015).

[b5] Schwindt M. A. . Unique and efficient synthesis of [2S-(2R*,3S*,4R*)]-2-amino-1-cyclohexyl-6-methyl-3,4-heptanediol, a popular C-terminal component of many renin inhibitors. J Org Chem 61, 9564–9568 (1996).

[b6] Effenberger F., Gutterer B. & Jäger J. Stereoselective synthesis of (1R)- and (1R,2S)-1-aryl-2-alkylamino alcohols from (R)-cyanohydrins. Tetrahedron: Asymmetry 8, 459–467 (1997).

[b7] Lapworth A. XCVI.-Reactions involving the addition of hydrogen cyanide to carbon compounds. J Chem Soc Trans 83, 995–1005 (1903).

[b8] Evans D., Truesdale L. & Carroll G. Cyanosilylation of aldehydes and ketones. A convenient route to cyanohydrin derivatives. J Chem Soc Chem Comm 1, 55–56 (1973).

[b9] Golinski M., Brock C. P. & Watt D. S. Addition of tert-butyldimethyl- or tert-butyldiphenylsilyl cyanide to hindered ketones. J Org Chem 58, 159–164 (1993).

[b10] Baeza A., Casas J., Nájera C., Sansano J. M. & Saá J. M. Enantioselective synthesis of cyanohydrin O-phosphates mediated by the bifunctional catalyst binolam–AlCl. Angew Chem Int Ed 42, 3143–3146 (2003). 10.1002/anie.200351552 12866102

[b11] Khan N. H. . Asymmetric synthesis of O-acetylcyanohydrins by reaction of aldehydes with NaCN/KCN catalyzed by recyclable chiral dimeric titanium(IV)/vanadium(V) salen complexes. Eur J Org Chem 71, 3175–3180 (2006).

[b12] Kikukawa Y. . Cyanosilylation of carbonyl compounds with trimethylsilyl cyanide catalyzed by an yttrium-pillared silicotungstate dimer. Angew Chem 124, 3746–3750 (2012). 10.1002/anie.201200486 22378706

[b13] Zhang Z. . Insight into the catalytic properties and applications of metal-organic frameworks in the cyanosilylation of aldehydes. RSC Adv 5, 79355–79360 (2015).

[b14] Cui X., Xu M. C., Zhang L. J., Yao R. X. & Zhang X. M. Solvent-free heterogeneous catalysis for cyanosilylation in a dynamic cobalt-MOF. Dalton Trans 44, 12711–12716 (2015). 26086918 10.1039/c5dt01456e

[b15] Hamashima Y., Sawada D., Nogami H., Kanai M. & Shibasaki M. Highly enantioselective cyanosilylation of aldehydes catalyzed by a Lewis acid–Lewis base bifunctional catalyst. Tetrahedron 57, 805–814 (2001).

[b16] Tian S.-K., Hong R. & Deng L. A Catalytic asymmetric cyanosilylation of ketones with chiral Lewis base. J Am Chem Soc 125, 9900–9901 (2003). 12914434 10.1021/ja036222p

[b17] Karimi B. & Ma’Mani L. A highly efficient and recyclable silica-based scandium(III) interphase catalyst for cyanosilylation of carbonyl compounds. Org Lett 6, 4813–4815 (2004). 15606073 10.1021/ol0482083

[b18] Kurono N., Yamaguchi M., Suzuki K. & Ohkuma T. Lithium chloride: an active and simple catalyst for cyanosilylation of aldehydes and ketones. J Org Chem 70, 6530–6532 (2005). 16050725 10.1021/jo050791t

[b19] Matsukawa S., Sekine I. & Iitsuka A. Tris (2,4,6-trimethoxyphenyl) phosphine (TTMPP): efficient catalysts for the cyanosilylation and cyanocarbonation of aldehydes and ketones. Molecules 14, 3353–3359 (2009). 19783929 10.3390/molecules14093353 PMC6254716

[b20] North M. A bimetallic titanium catalyst for the enantioselective cyanation of aldehydes based on cooperative catalysis. Angew Chem Int Ed 49, 8079–8081 (2010). 10.1002/anie.201003014 20809557

[b21] Zhang Z., Wang Z., Zhang R. & Ding K. An efficient titanium catalyst for enantioselective cyanation of aldehydes: cooperative catalysis. Angew Chem Int Ed 49, 6746–6750 (2010). 10.1002/anie.201002127 20632347

[b22] Benaglia M. & Rossi S. Chiral phosphine oxides in present-day organocatalysis. Org Biomol Chem 8, 3824–3830 (2010). 20567785 10.1039/c004681g

[b23] Lacour M. A., Rahier N. J. & Taillefer M. Mild and efficient trimethylsilylcyanation of ketones catalysed by PNP chloride. Chem Eur J 17, 12276–12279 (2011). 21922572 10.1002/chem.201101195

[b24] North M., Omedes-Pujol M. & Young C. Kinetics and mechanism of the racemic addition of trimethylsilyl cyanide to aldehydes catalysed by Lewis bases. Org Biomol Chem 10, 4289–4298 (2012). 22544042 10.1039/c2ob25188d

[b25] Gaunt M. J., Johansson C. C. C., McNally A. & Vo N. T. Enantioselective organocatalysis. Drug Discovery Today 12, 8–27 (2007). 17198969 10.1016/j.drudis.2006.11.004

[b26] Enders D., Niemeier O. & Henseler A. Organocatalysis by *N*-heterocyclic carbenes. Chem Rev 107, 5606–5655 (2007). 17956132 10.1021/cr068372z

[b27] MacMillan D. W. C. The advent and development of organocatalysis. Nature 455, 304–308 (2008). 18800128 10.1038/nature07367

[b28] Zhang Z., Lippert K. M., Hausmann H., Kotke M. & Schreiner P. R. Cooperative thiourea–bronsted acid organocatalysis: enantioselective cyanosilylation of aldehydes with TMSCN. J Org Chem 76, 9764–9776 (2011). 22011108 10.1021/jo201864e

[b29] Wen Y., Liang M., Wang Y., Ren W. & Lü X. Perfectly green organocatalysis: quaternary ammonium base triggered cyanosilylation of aldehydes. Chin J Chem 30, 2109–2114 (2012).

[b30] Marcia de Figueiredo R. & Christmann M. Organocatalytic synthesis of drugs and bioactive natural products. Eur J Org Chem 2007, 2575–2600 (2007).

[b31] Fukuda Y., Maeda Y., Ishii S., Kondo K. & Aoyama T. An *N*-heterocyclic carbene as a nucleophilic catalyst for cyanosilylation of aldehydes. Synthesis 2006, 589–590 (2006).

[b32] Song J. J. . Activation of TMSCN by *N*-heterocyclic carbenes for facile cyanosilylation of carbonyl compounds. J Org Chem 71, 1273–1276 (2006). 16438554 10.1021/jo052206u

[b33] Suzuki Y., Bakar M. D. A., Muramatsu K. & Sato M. Cyanosilylation of aldehydes catalyzed by *N*-heterocyclic carbenes. Tetrahedron 62, 4227–4231 (2006).

[b34] Marion N., Díez-González S. & Nolan S. P. *N*-heterocyclic carbenes as organocatalysts. Angew Chem Int Ed 46, 2988–3000 (2007). 10.1002/anie.200603380 17348057

[b35] Kano T., Sasaki K., Konishi T., Mii H. & Maruoka K. Highly efficient trialkylsilylcyanation of aldehydes, ketones and imines catalyzed by a nucleophilic *N*-heterocyclic carbene. Tetrahedron Lett 47, 4615–4618 (2006).

[b36] Fredlake C. P., Crosthwaite J. M., Hert D. G., Aki S. N. V. K. & Brennecke J. F. Thermophysical properties of imidazolium-based ionic liquids. J Chem Eng Data 49, 954–964 (2004).

[b37] Chiappe C. & Pieraccini D. Ionic liquids: solvent properties and organic reactivity. J Phys Org Chem 18, 275–297 (2005).

[b38] Zhang S., Sun N., He X., Lu X. & Zhang X. Physical properties of ionic liquids: database and evaluation. J Phys Chem Ref Data 35, 1475–1517 (2006).

[b39] Gao H., Guo C., Xing J., Zhao J. & Liu H. Extraction and oxidative desulfurization of diesel fuel catalyzed by a bronsted acidic ionic liquid at room temperature. Green Chem 12, 1220–1224 (2010).

[b40] Zhang P., Qiao Z. A., Jiang X., Veith G. M. & Dai S. Nanoporous ionic organic networks: stabilizing and supporting gold nanoparticles for catalysis. Nano Lett 15, 823–828 (2015). 25625306 10.1021/nl504780j

[b41] Giacalone F. & Gruttadauria M. Covalently supported ionic liquid phases: an advanced class of recyclable catalytic systems. Chem Cat Chem 8, 664–684 (2016).

[b42] Sun N. . Complete dissolution and partial delignification of wood in the ionic liquid 1-ethyl-3-methylimidazolium acetate. Green Chem 11, 646–655 (2009).

[b43] Olivier-Bourbigou H., Magna L. & Morvan D. Ionic liquids and catalysis: Recent progress from knowledge to applications. Appl Catal A 373, 1–56 (2010).

[b44] Shen Z. L., Ji S. J. & Loh T. P. Ionic liquid [omim][PF 6] as an efficient and recyclable reaction media for the cyanosilylation of aldehydes without Lewis acid or any special activation. Tetrahedron Lett 46, 3137–3139 (2005).

[b45] YeonáRyu K. & HwanáPark J. A dream combination for catalysis: highly reactive and recyclable scandium (III) triflate-catalyzed cyanosilylations of carbonyl compounds in an ionic liquid. Green Chem 11, 946–948 (2009).

[b46] Baleizão C., Gigante B., García H. & Corma A. Chiral vanadyl salen complex anchored on supports as recoverable catalysts for the enantioselective cyanosilylation of aldehydes. Comparison among silica, single wall carbon nanotube, activated carbon and imidazolium ion as support. Tetrahedron 60, 10461–10468 (2004).

[b47] Sans V. . Polymer-supported ionic-liquid-like phases (SILLPs): transferring ionic liquid properties to polymeric matrices. Chem Eur J 17, 1894–1906 (2011). 21274940 10.1002/chem.201001873

[b48] Martin S. . Supported ionic liquid-like phases as organocatalysts for the solvent-free cyanosilylation of carbonyl compounds: from batch to continuous flow process. Green Chem 16, 1639–1647 (2014).

[b49] Yang Q. . One of the distinctive properties of ionic liquids over molecular solvents and inorganic salts: enhanced basicity stemming from the electrostatic environment and “free” microstructure. J Phys Chem B 118, 3682–3688 (2014). 24620779 10.1021/jp500790r

[b50] North M. Synthesis and applications of non-racemic cyanohydrins. Tetrahedron: Asymmetry 14, 147–176 (2003).

[b51] Holmes I. P. & Kagan H. B. The asymmetric addition of trimethylsilylcyanide to aldehydes catalysed by anionic chiral nucleophiles. Part 1. Tetrahedron Lett 41, 7453–7456 (2000).

[b52] Holmes I. P. & Kagan H. B. The asymmetric addition of trimethylsilylcyanide to aldehydes catalysed by anionic chiral nucleophiles. Part 2. Tetrahedron Lett 41, 7457–7460 (2000).

[b53] Chuit C., Corriu R. J., Reye C. & Young J. C. Reactivity of penta-and hexacoordinate silicon compounds and their role as reaction intermediates. Chem Rev 93, 1371–1448 (1993).

[b54] Holmes R. R. Comparison of phosphorus and silicon: hypervalency, stereochemistry, and reactivity. Chem Rev 96, 927–950 (1996). 11848776 10.1021/cr950243n

[b55] Ott L. S., Cline M. L., Deetlefs M., Seddon K. R. & Finke R. G. Nanoclusters in ionic liquids: evidence for *N*-heterocyclic carbene formation from imidazolium-based ionic liquids detected by ^2^H NMR. J Am Chem Soc 127, 5758–5759 (2005). 15839652 10.1021/ja0423320

[b56] Kelemen Z., Holloczki O., Nagy J. & Nyulaszi L. An organocatalytic ionic liquid. Org Biomol Chem 9, 5362–5364 (2011). 21701727 10.1039/c1ob05639e

[b57] Rodriguez H., Gurau G., Holbrey J. D. & Rogers R. D. Reaction of elemental chalcogens with imidazolium acetates to yield imidazole-2-chalcogenones: direct evidence for ionic liquids as proto-carbenes. Chem Commun 47, 3222–3224 (2011). 10.1039/c0cc05223j 21301714

[b58] Hollóczki O. . Carbene formation in ionic liquids: spontaneous, induced, or prohibited? J Phys Chem B 117, 5898–5907 (2013). 23566121 10.1021/jp4004399

[b59] Coupillaud P.. Poly (ionic liquid) s based on imidazolium hydrogen carbonate monomer units as recyclable polymer‐supported *N*‐heterocyclic carbenes: use in organocatalysis. J Polym Sci Part A: Polym Chem 51, 4530–4540 (2013).

[b60] Liu D. & Chen E. Y. X. Organocatalysis in biorefining for biomass conversion and upgrading. Green Chem 16, 964–981 (2014).

[b61] Thomas M., Brehm M., Hollóczki O. & Kirchner B. How can a carbene be active in an ionic liquid? Chem Eur J 20, 1622–1629 (2014). 24375892 10.1002/chem.201303329

[b62] Clough M. T., Geyer K., Hunt P. A., Mertes J. & Welton T. Thermal decomposition of carboxylate ionic liquids: trends and mechanisms. Phys Chem Chem Phys 15, 20480–20495 (2013). 24173605 10.1039/c3cp53648c

