# Supplementary material for: 1-Ethyl-3-methylimidazolium acetate as a highly efficient organocatalyst for cyanosilylation of carbonyl compounds with trimethylsilyl cyanide
Source: Sci Rep. 2017 Feb 15;7:42699. doi: 10.1038/srep42699 (PMC5309884; doi:10.1038/srep42699)
Supplement: Supplementary Information [file srep42699-s1.pdf]

Supplementary Information

1-Ethyl-3-methylimidazolium acetate as a highly efficient organocatalyst for cyanosilylation of carbonyl compounds with trimethylsilyl cyanide

Bakhtar Ullah, Jingwen Chen, Zhiguo Zhang,* Huabin Xing, Qiwei Yang, Zongbi Bao, Qilong Ren

Key Laboratory of Biomass Chemical Engineering of Ministry of Education, College of Chemical and Biological Engineering, Zhejiang University, Hangzhou 310027, P. R. China.

Contents

1. Materials and instruments. S2
2. Blank experiments for all the screened substrates. S2
3. ^{1}H NMR spectral data of the products/mixtures..S4
4. ^{1}H NMR measurement of TMSCN with or without [EMIM]OAc (1a). S13
5. References...S13

EXPERIMENTAL SECTION

1. Materials and instruments.

For the catalytic cyanosilylation of carbonyl compounds studies, all necessary starting materials including imidazolium based ionic liquids (ILs), carbonyl compounds and solvents etc. were purchased from commercial suppliers and were used without further purification unless otherwise stated. Trimethylsilyl cyanide (TMSCN) and other carbonyl compounds were purchased from Aladdin. The aldehydes and TMSCN were freshly distilled at ambient pressure and then stored under nitrogen atmosphere. All screened imidazolium based ILs were diluted in CH_3CN before using as catalysts in the cyanosilylation of carbonyl compounds with TMSCN. Solvents used for chromatography or for optimization of reaction conditions, were of reagent grade.

After optimization of conditions, all cyanosilylation reactions were carried out under solvent-free medium at room temperature. The conversions were monitored by TLC with silica gel-coated glass plates using UV light to envision the progress of the reaction. Flash column chromatography was performed using Macherey-Nagel silica gel (200–300 mesh). All NMR experiments were recorded on Agilent 600 MHz or Bruker Advance 400 MHz NMR spectrometers. Data are reported as follows: chemical shift (ppm), multiplicity (s = singlet, d = doublet, t = triplet, q = quartet, dd = doublet of doublets, ddd = doublet of doublets of doublets, td = triplet of doublets, dt = doublet of triplets, m = multiplet), coupling constants (Hz), and integration. The ^1H chemical shifts were referred to TMS (internal standard) of the solvent ($CDCl_3$) at 0.00 ppm.

2. Blank experiments for all the screened substrates.

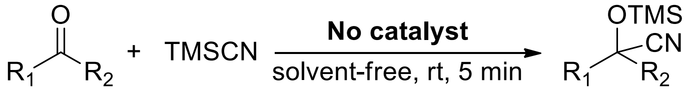

10 11

**Entry**	**R^1**	**R^2**	**Yield/%^{b}**
1	Ph	H	Trace
2	*p*-Cl-Ph	H	24
3	*m*-F-Ph	H	25
4	*m*-MeO-Ph	H	0
5	Cinnamyl	H	Trace
6	2-Furyl	H	Trace
7	Cyclohexyl	H	30
8	*i*-Pr	H	33
9	Octyl	H	32
10	Ph	Me	0
11	*p*-Cl-Ph	Me	0
12	*o*-F-Ph	Me	0
13	*p*-Br-Ph	Me	0
14	*p*- *i*-Pr	Me	0
15	*p*-Me-Ph	Me	0
16	*n*-Bu	Me	0
17	Cyclohexanone	-	0
18	Ph	Ph	0

Supplementary Table S1. Blank experiments for all the screened substrates are given below in Table S1.[a] [a]The reaction was carried out with various carbonyl compounds (1.0 mmol) and TMSCN (1.2 mmol) without catalyst for 5 minutes under neat conditions at room temperature. [b]Determined by ^{1}H NMR.

3. ^{1}H NMR spectral data of the products/mixtures.

2-Phenyl-2-trimethylsilyloxyacetonitrile (Table 3, entry 1)

^{1}H NMR (400 MHz, CDCl3) δ 10.00 (s, 1H), 7.48 – 7.36 (m, 5H, aromatics), 5.49 (s, 1H, CHCN), 0.22 (s, 9H, OTMS).

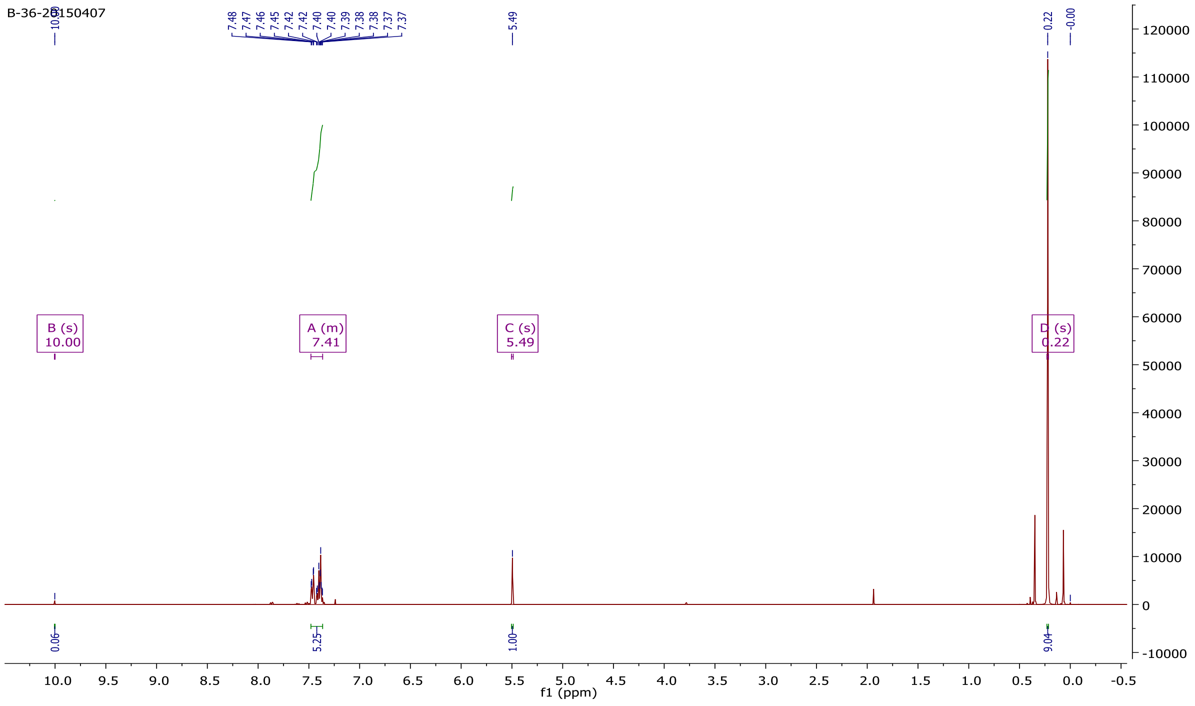

2-(4-chlorophenyl)-2-trimethylsilyloxyacetonitrile (Table 3, entry 2)

^{1}H NMR (600 MHz, $CDCl_3$) δ 7.44 – 7.37 (m, 4H), 5.47 (s, 1H), 0.24 (s, 9H).

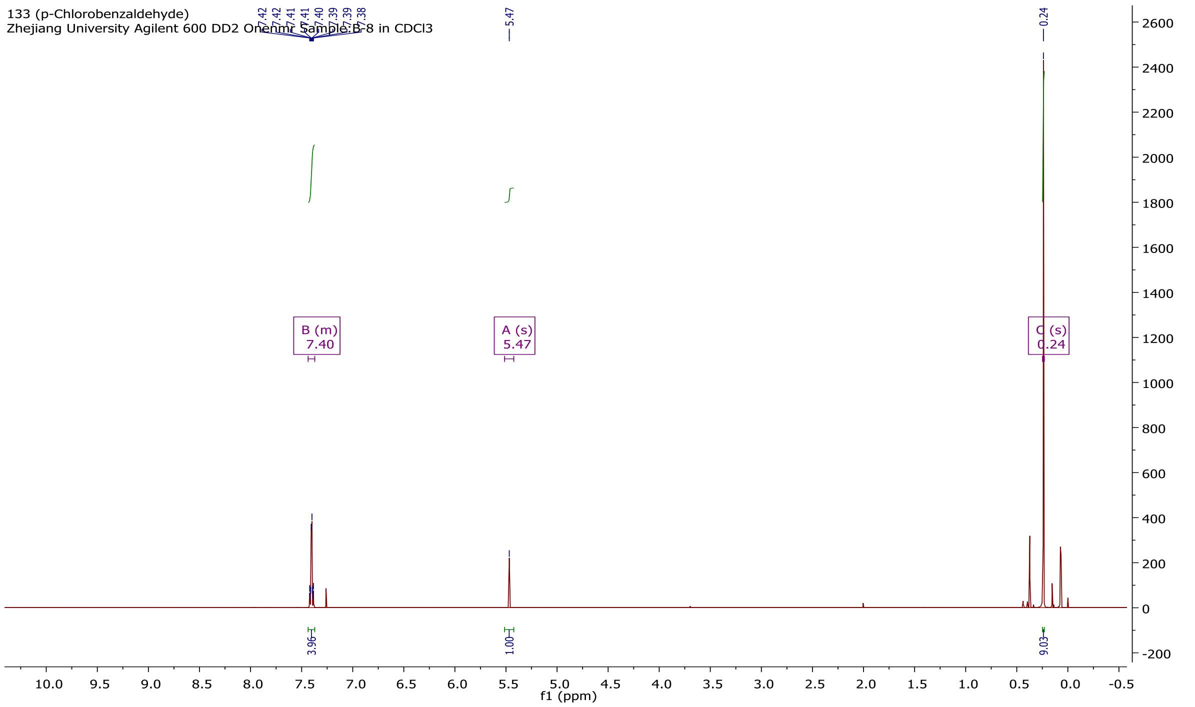

2-(3-fluorophenyl)-2-trimethylsilyloxyacetonitrile (Table 3, entry 3)

^{1}H NMR (600 MHz, $CDCl_3$) δ 7.41 – 7.36 (m, 1H), 7.26 – 7.19 (m, 2H), 7.08 (td, J = 8.3, 2.5 Hz, 1H), 5.49 (s, 1H), 0.25 (s, 9H).

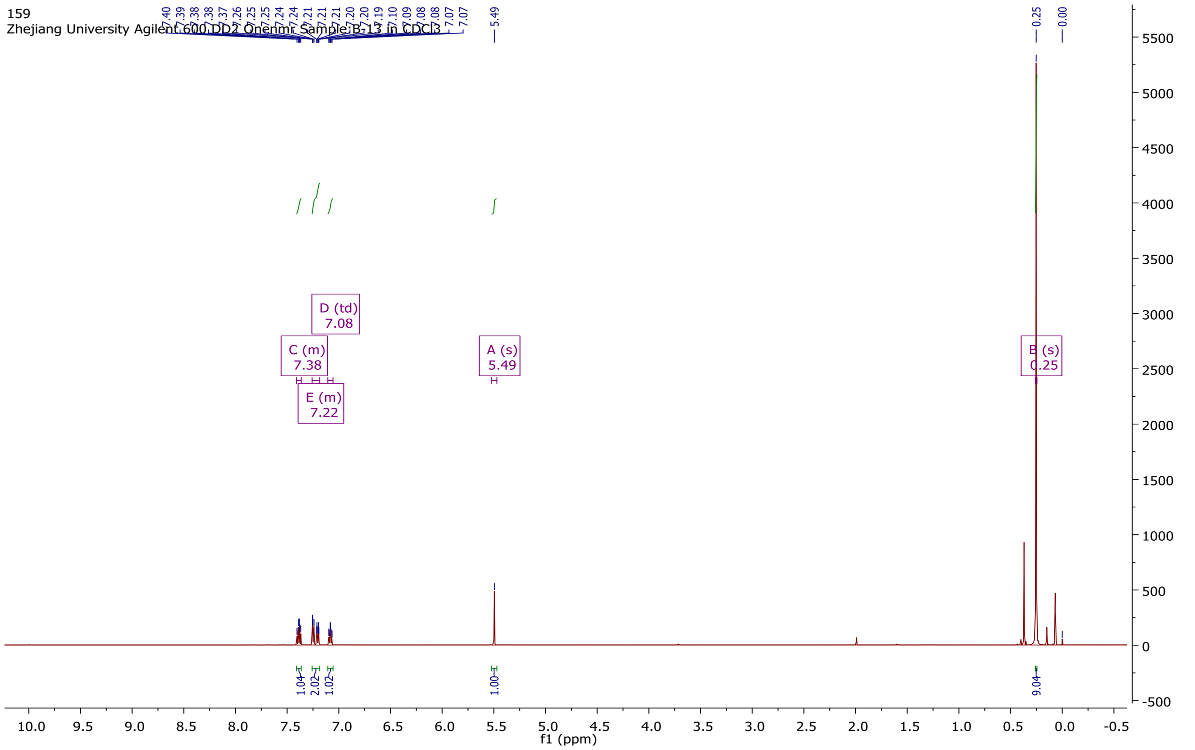

2-(3-methoxyphenyl)-2-trimethylsilyloxyacetonitrile (Table 3, entry 4)

^{1}H NMR (400 MHz, $CDCl_3$) δ 7.31 (t, J = 7.9 Hz, 1H), 7.03 (dd, J = 9.0, 1.6 Hz, 2H), 6.91 (ddd, J = 8.4, 2.5, 0.8 Hz, 1H), 5.47 (s, 1H), 3.81 (s, 3H), 0.23 (s, 9H).

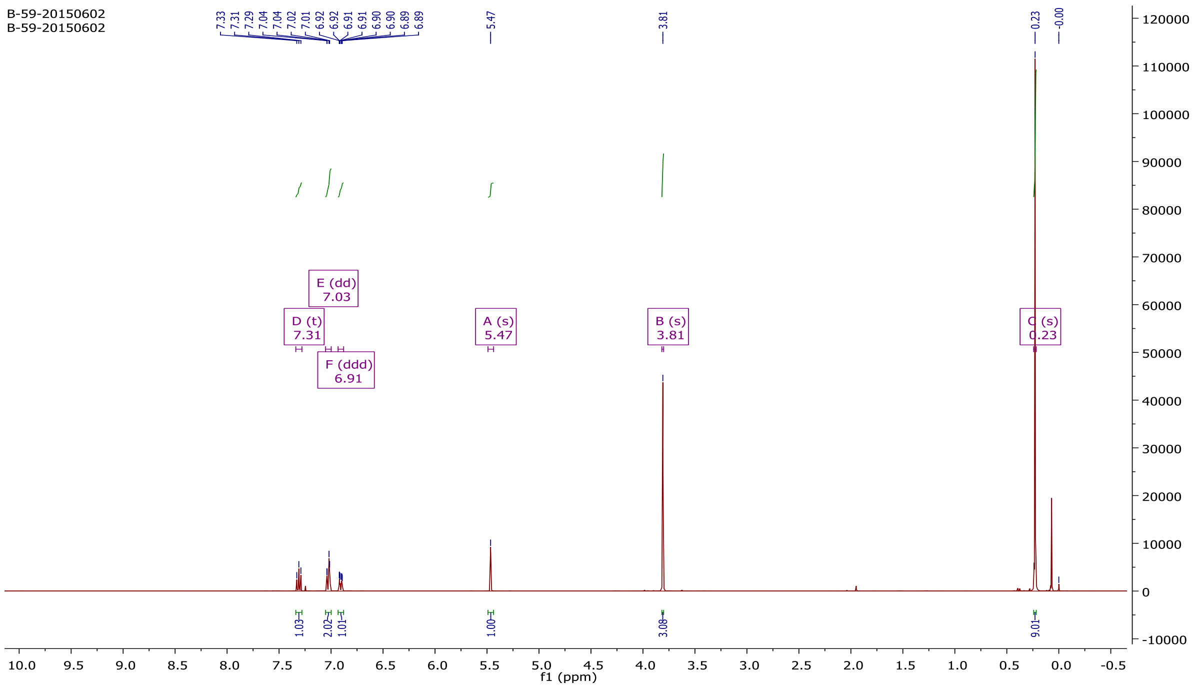

4-Phenyl-2-(trimethylsilyloxy)but-3-enenitrile (Table 3, entry 5)

^{1}H NMR (400 MHz, $CDCl_3$) δ 7.41 – 7.29 (m, 5H), 6.80 (dd, J = 15.8, 0.9 Hz, 1H), 6.18 (dd, J = 15.8, 6.0 Hz, 1H), 5.10 (dd, J = 6.0, 1.3 Hz, 1H), 0.25 (s, 9H).

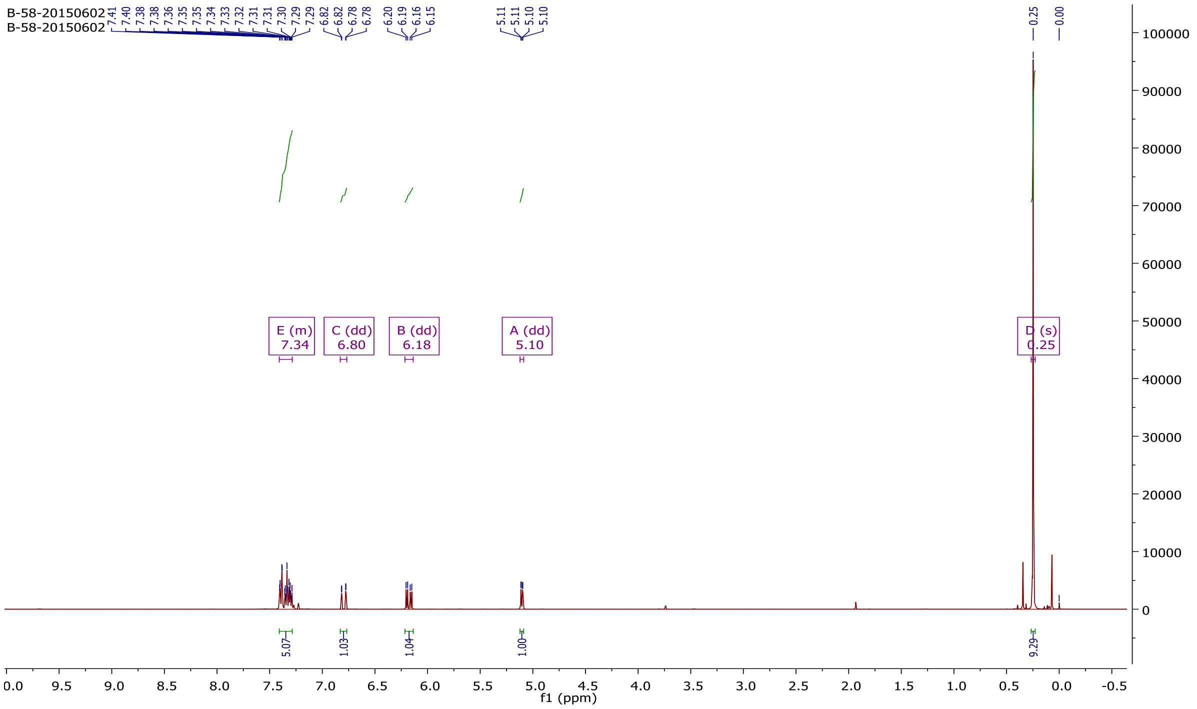

2-furanyl-2-trimethylsilyloxyacetonitrile (Table 3, entry 6)

^{1}H NMR (400 MHz, $CDCl_3$) δ 9.65 (s, 1H), 7.46 (dd, J = 1.8, 0.7 Hz, 1H), 6.54 (d, J = 3.3 Hz, 1H), 6.40 (dd, J = 3.3, 1.9 Hz, 1H), 5.55 (s, 1H), 0.19 (s, 9H).

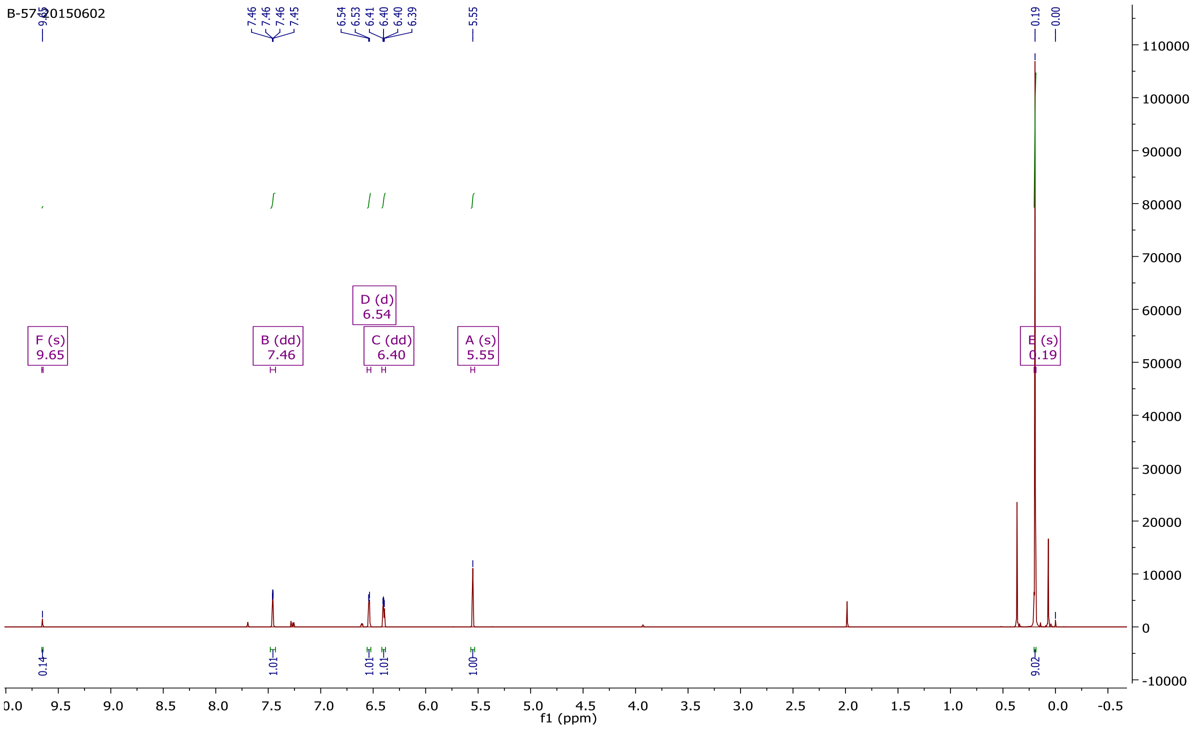

2-cyclohexyl-2-trimethylsilyloxyacetonitrile (Table 3, entry 7)

^{1}H NMR (400 MHz, $CDCl_3$) δ 4.15 (d, J = 6.3 Hz, 1H), 1.90 – 1.60 (m, 6H), 1.31 – 1.00 (m, 5H), 0.20 (s, 9H).

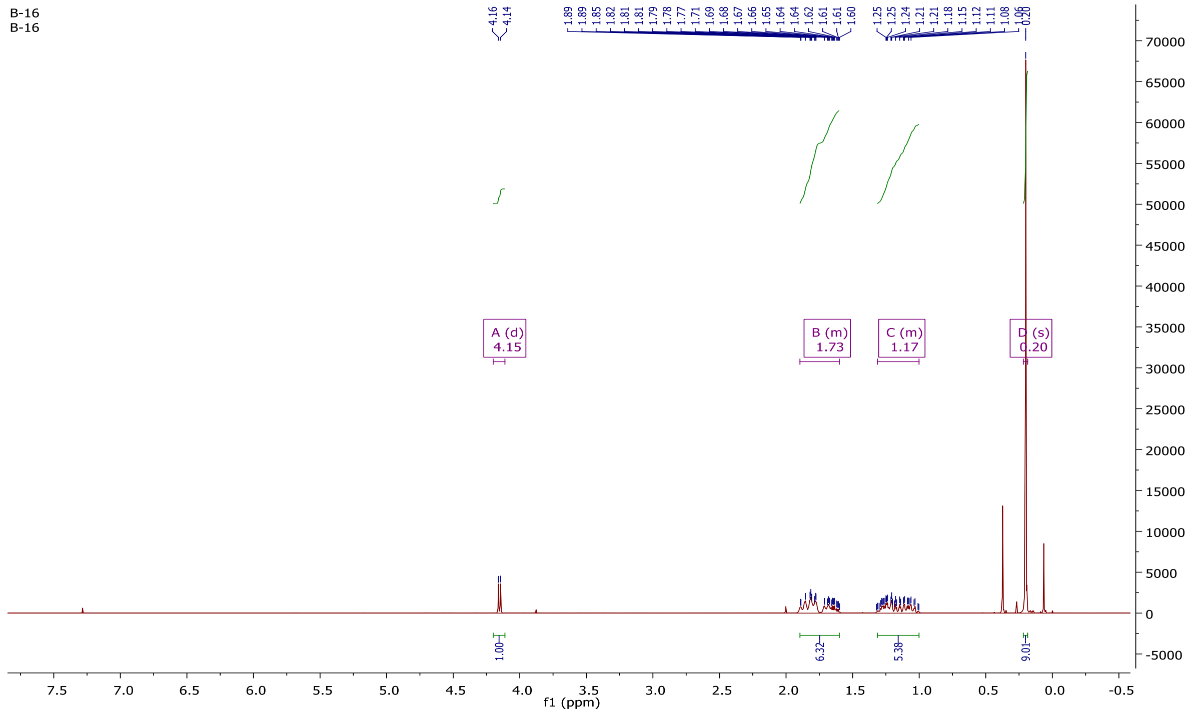

3-methyl-2-trimethylsilyloxybutanenitrile (Table 3, entry 8)

^{1}H NMR (400 MHz, $CDCl_3$) δ 4.17 (d, J = 5.8 Hz, 1H), 2.02 – 1.90 (m, 1H), 1.03 (dd, J = 12.2, 6.8 Hz, 6H), 0.21 (s, 9H).

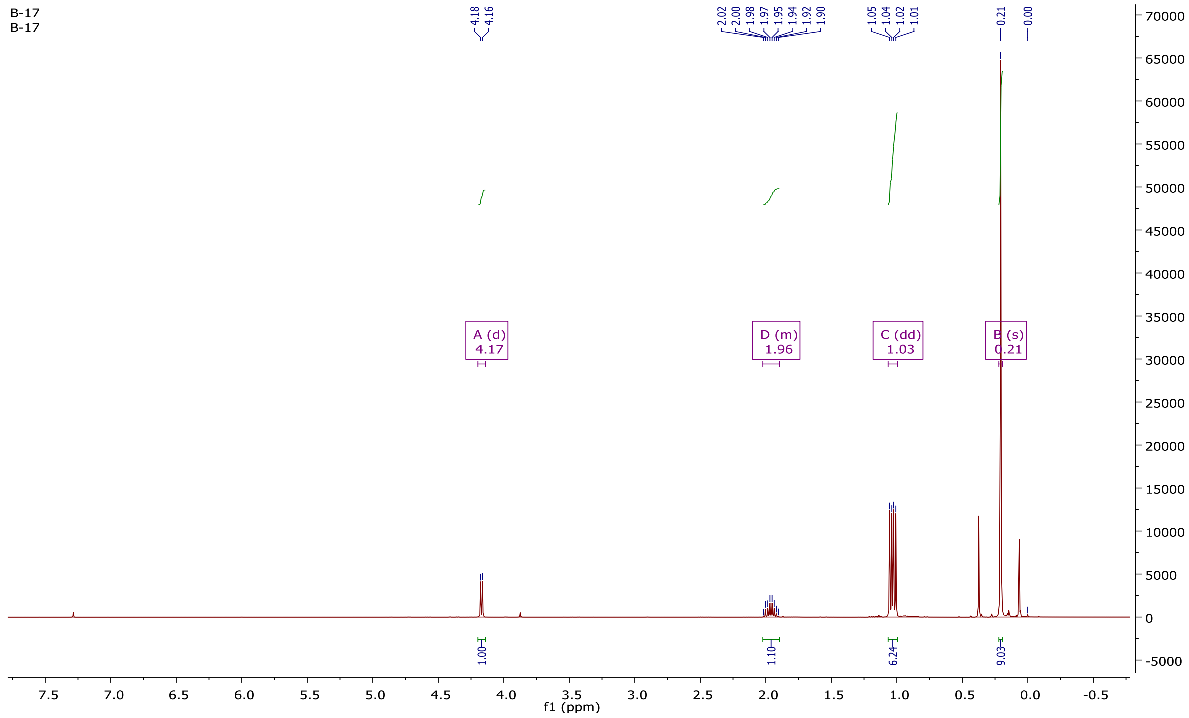

2-trimethylsilyloxynonanenitrile (Table 3, entry 9)

^{1}H NMR (400 MHz, $CDCl_3$) δ 4.39 (t, J = 6.6 Hz, 1H), 1.81 – 1.74 (m, 2H), 1.47 – 1.27 (m, 10H), 0.89 (t, J = 6.9 Hz, 3H), 0.21 (s, 9H).

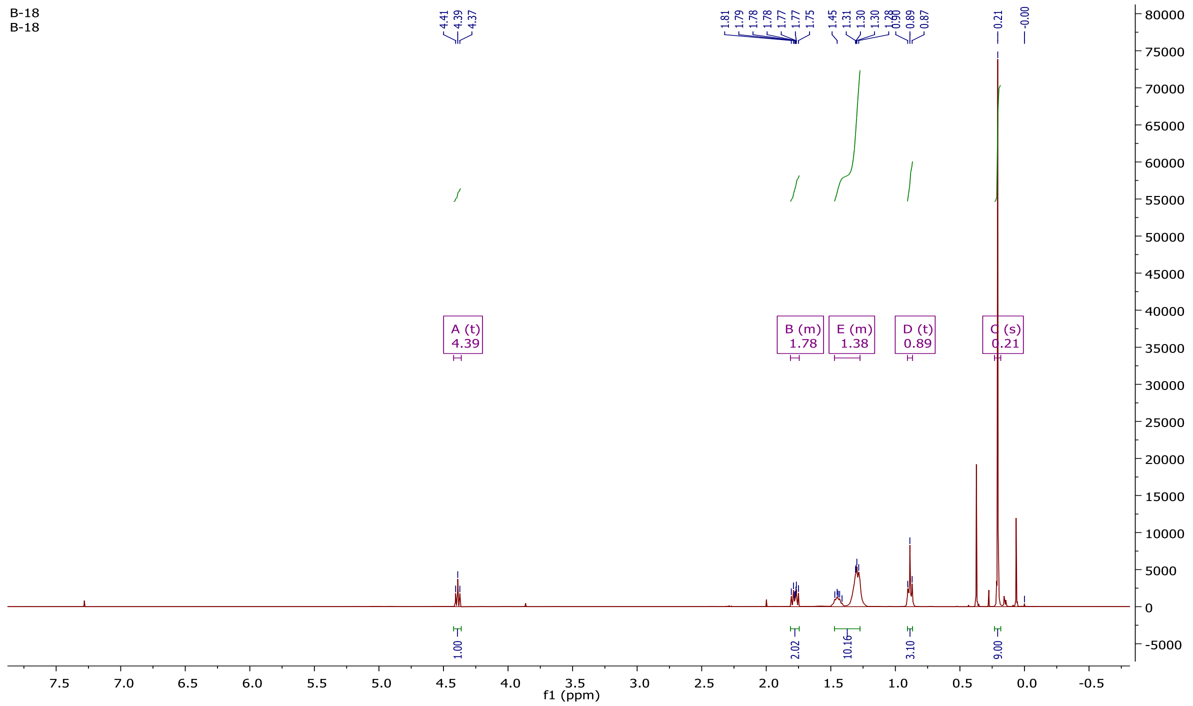

2-phenyl-2-trimethylsilyloxypropionitrile (Table 4, entry 1):

^{1}H NMR (400 MHz, $CDCl_3$): δ 7.56-7.53 (dt, 2H, aromatics), 7.42-7.33 (m, 3H, aromatics), 1.86 (s, 3H, $CHCH_3$), 0.17 (s, 9H, OTMS).

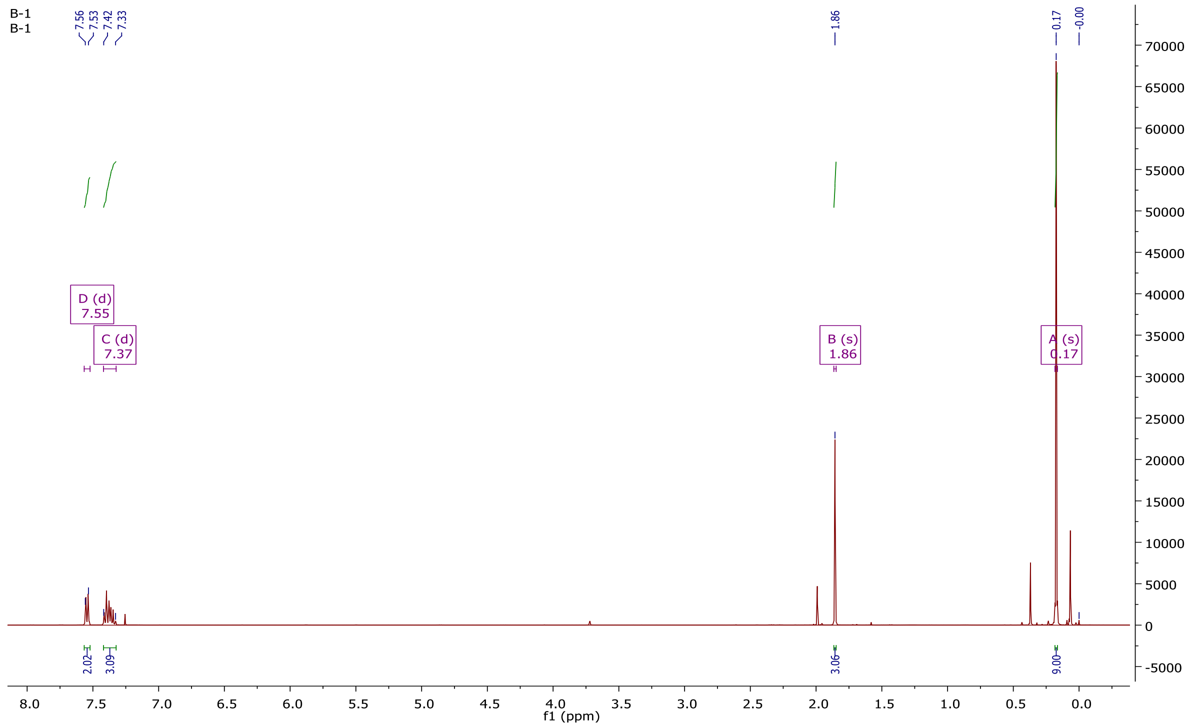

2-(4-chlorophenyl)-2-trimethylsilyloxypropanenitrile (Table 4, entry 2):

^{1}H NMR (400 MHz, $CDCl_3$) δ 7.50 – 7.47 (m, 2H), 7.39 – 7.35 (m, 2H), 1.83 (s, 3H), 0.19 (s, 9H).

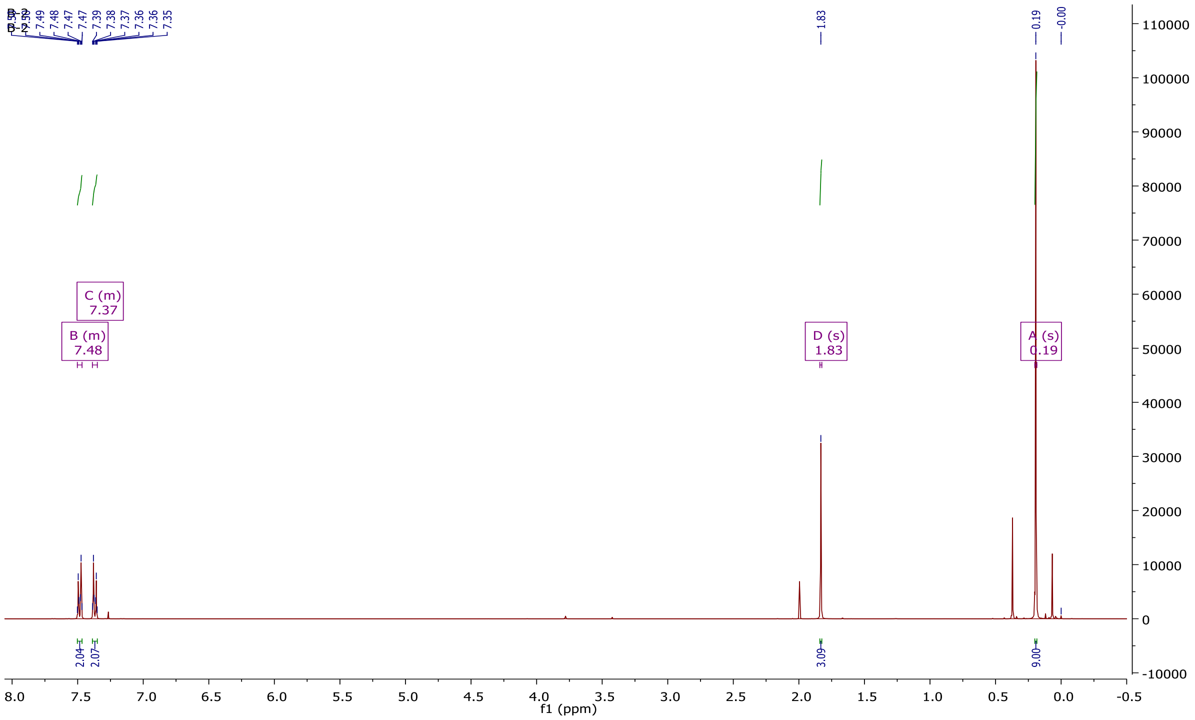

2-(2-fluorophenyl)-2-trimethylsilyloxypropanenitrile (Table 4, entry 3):

^{1}H NMR (400 MHz, $CDCl_3$): δ (ppm) = 0.26 (s, 9H, OTMS), 1.94 (s, 3H), 7.07-7.12 (m, 1H, aromatics), 7.16-7.20 (m, 1H, aromatics), 7.33-7.38 (m, 1H, aromatics), 7.56-7.60 (m, 1H, aromatics).

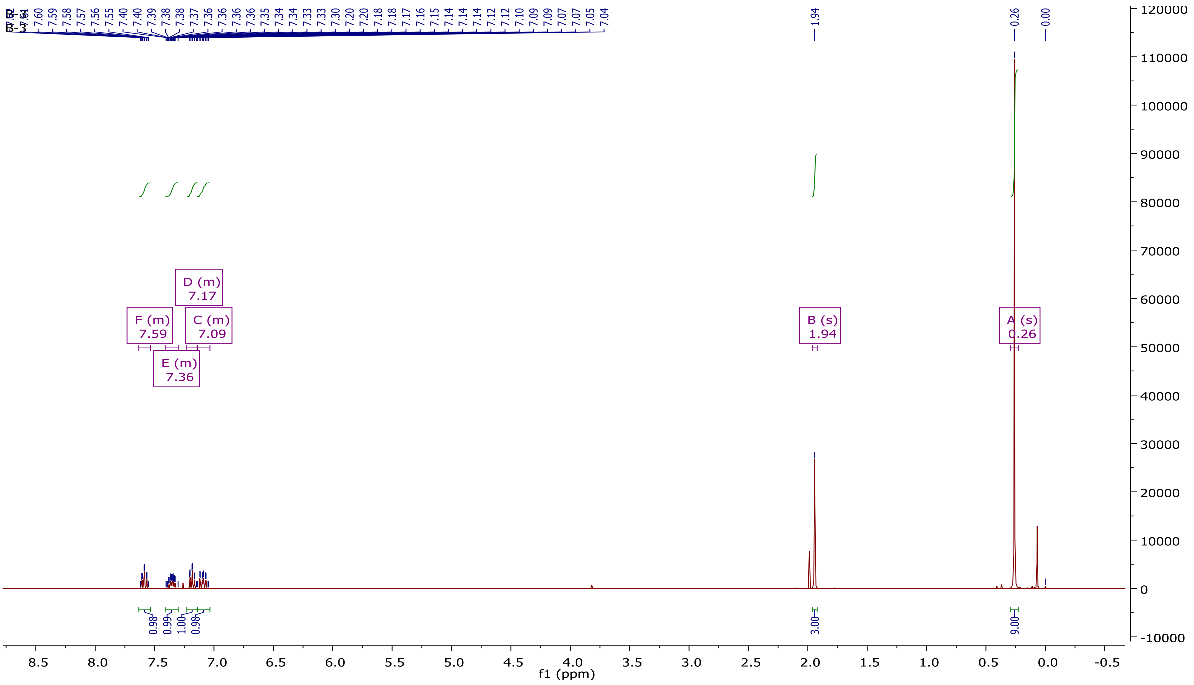

2-(4-bromophenyl)-2-trimethylsilyloxypropanenitrile (Table 4, entry 4):

^{1}H NMR (400 MHz, $CDCl_3$) δ 7.57 – 7.48 (m, 2H), 7.47 – 7.38 (m, 2H), 1.83 (s, 3H), 0.19 (s, 9H).

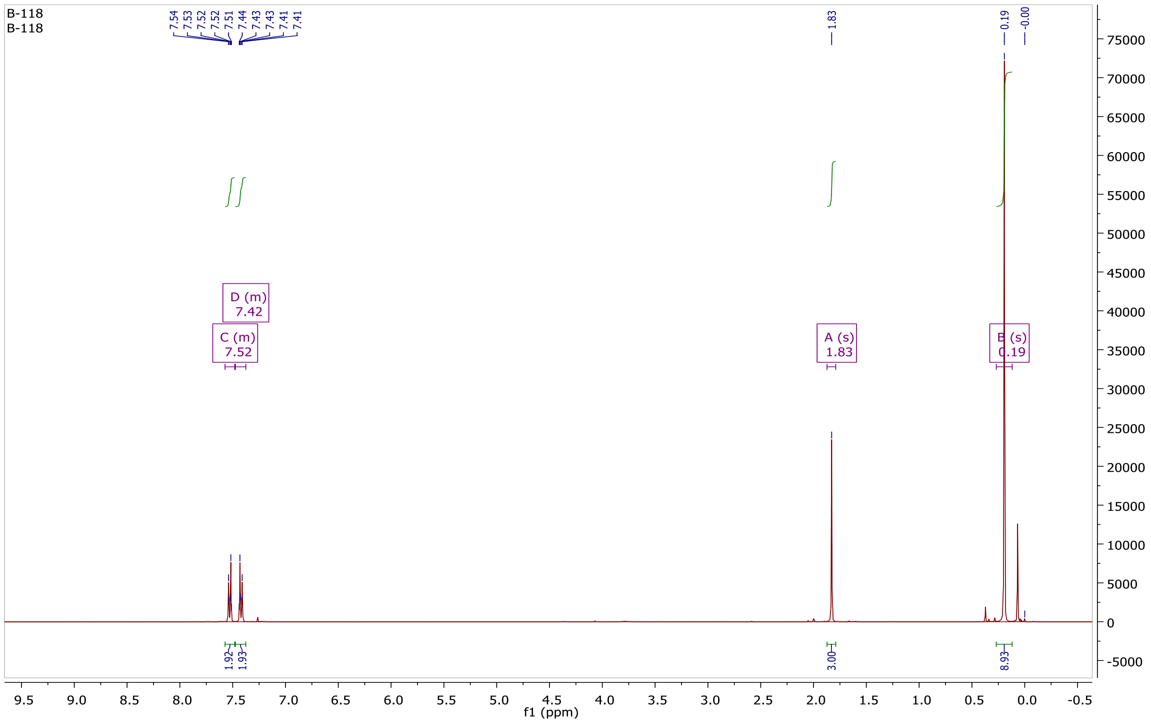

2-(4-isopropylphenyl)-2-trimethylsilyloxypropanenitrile (Table 4, entry 5):

^{1}H NMR (400 MHz, $CDCl_3$) δ 7.48 – 7.43 (m, 2H), 7.26 – 7.22 (m, 2H), 2.99 – 2.87 (m, 1H), 1.85 (s, 3H), 1.25 (d, J = 7.0 Hz, 6H), 0.17 (s, 9H).

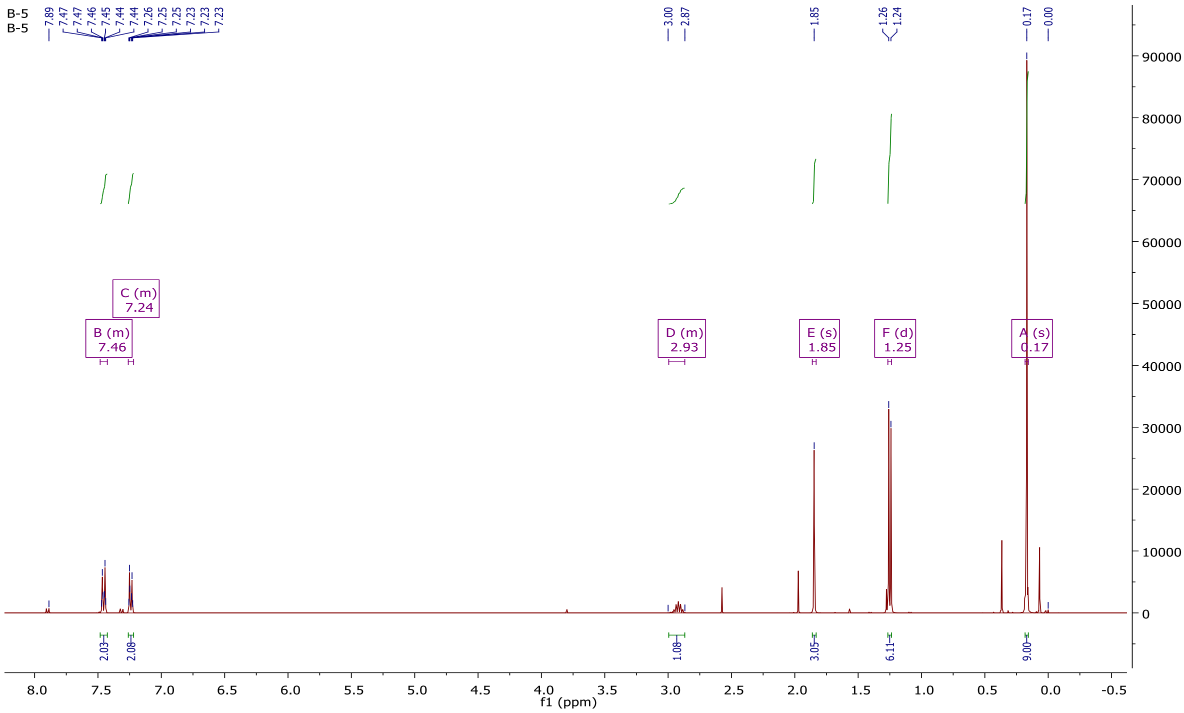

2-(4-methylphenyl)-2-trimethylsilyloxypropanenitrile (Table 4, entry 6):

^{1}H NMR (500 MHz, DMSO) δ 7.26 – 7.20 (m, 2H), 7.12 – 7.06 (m, 2H), 2.19 (s, 3H), 2.14 (s, 3H), 0.21 (s, 9H).

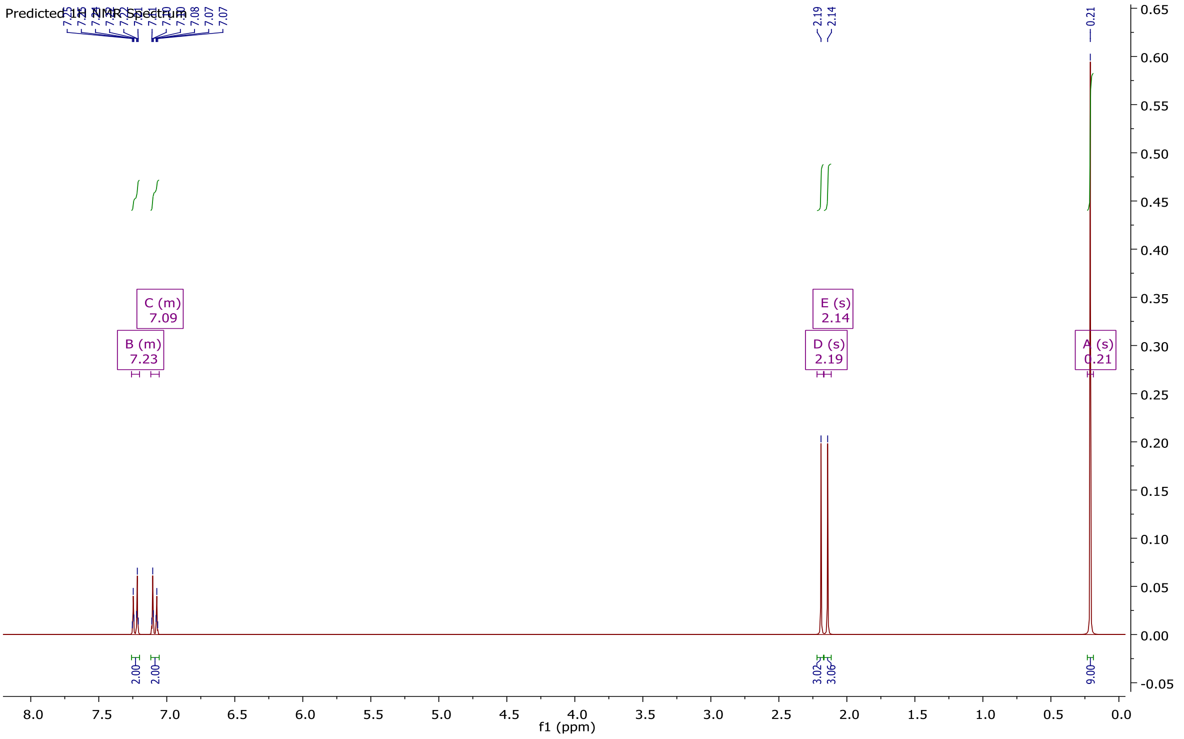

2-methyl-2-trimethylsilyloxyhexanecarbonitrile (Table 4, entry 7):

^{1}H NMR (400 MHz, $CDCl_3$) δ 1.78 – 1.64 (m, 2H), 1.56 (s, 3H), 1.54 – 1.30 (m, 4H), 0.93 (t, J = 7.2 Hz, 3H), 0.24 (s, 9H).

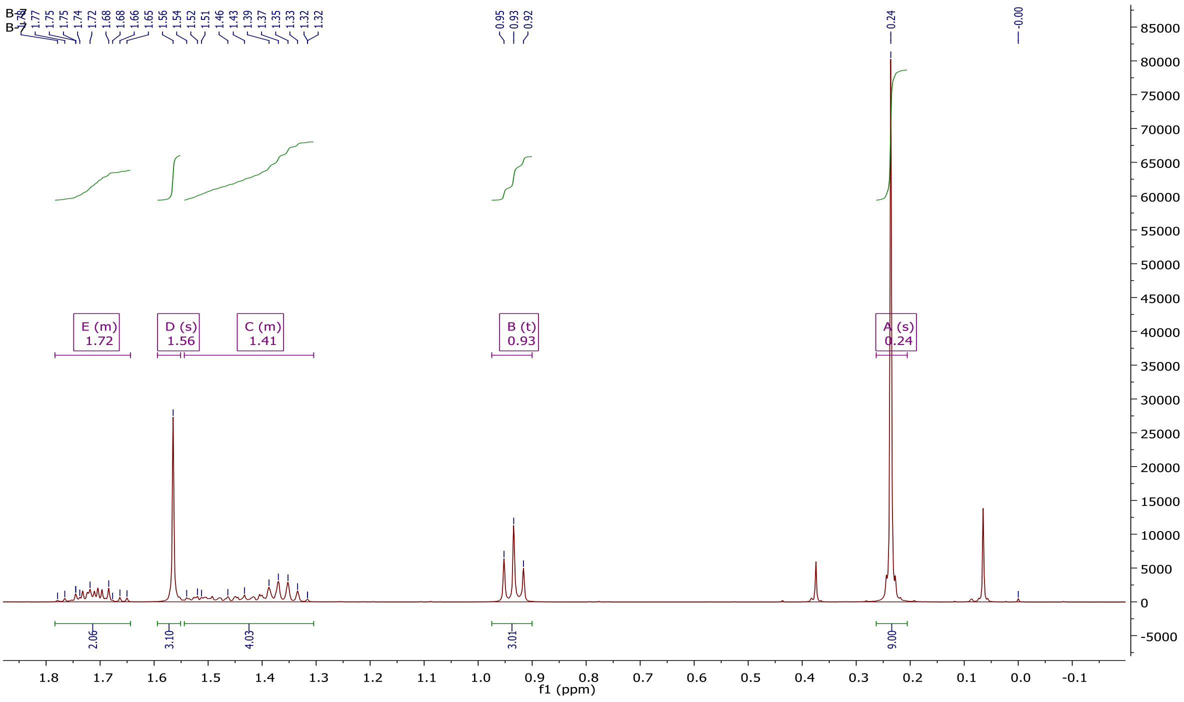

1-trimethylsilyloxy-1-cyclohexanecarbonitrile (Table 4, entry 8):

^{1}H NMR (400 MHz, $CDCl_3$) δ 1.78 – 1.64 (m, 2H), 1.56 (s, 3H), 1.54 – 1.30 (m, 4H), 0.93 (t, J = 7.2 Hz, 3H), 0.24 (s, 9H).

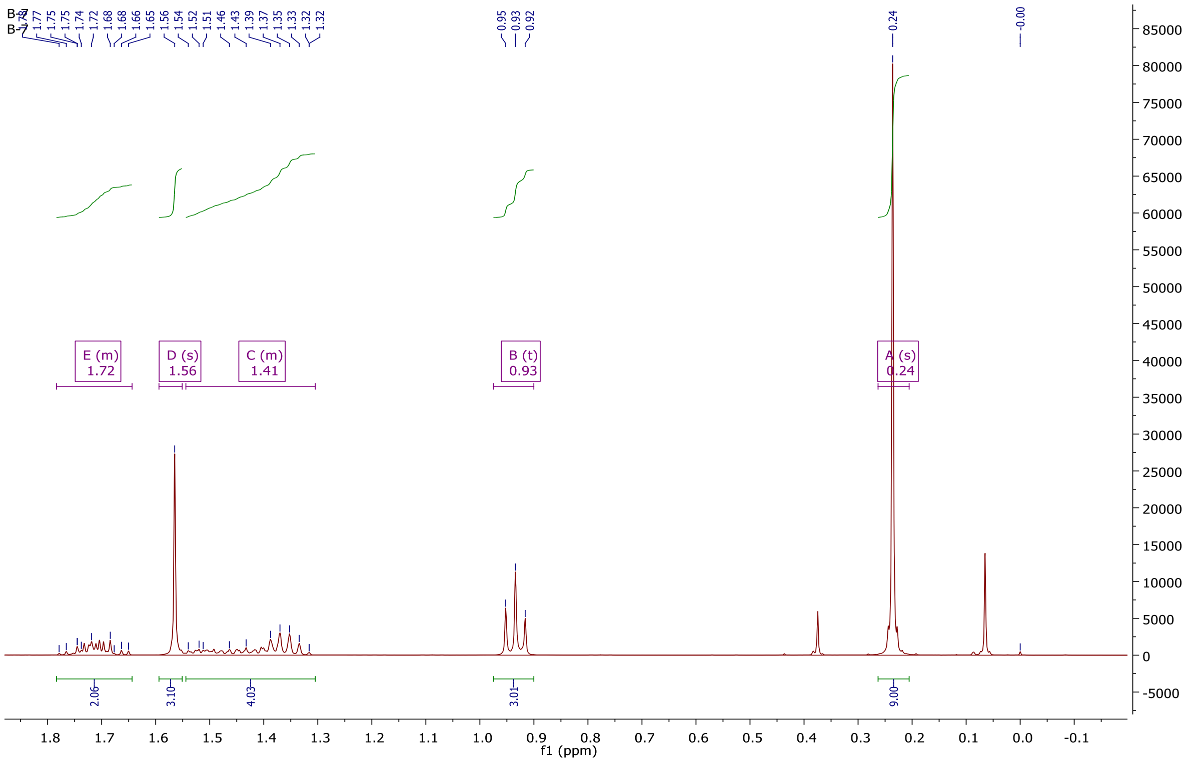

2,2-diphenyl-2-(trimethylsilyloxy)acetonitrile (Table 4, entry 9):

^{1}H NMR (400 MHz, $CDCl_3$) δ 7.55 – 7.48 (m, 4H), 7.39 – 7.29 (m, 6H), 0.15 (s, 9H).

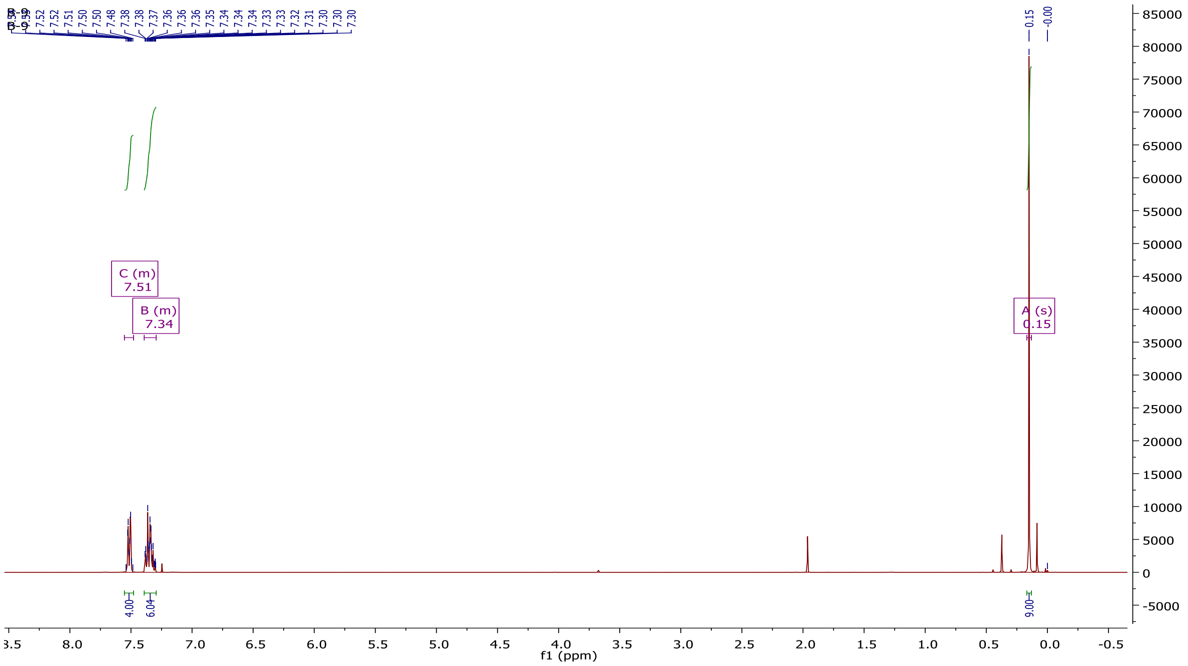

4. ^{1}H NMR measurement of TMSCN with or without [EMIM]OAc (1a).

The ^{1}H NMR spectra of both TMSCN [a], and a 1:1 mixture of TMSCN and **1a** [b], were evaluated in $CDCl_3$; the CH_3 peak of TMSCN observed at δ = 0.35 ppm was found to be shifted to δ = 0.15 ppm. The shift of the TMSCN peak in the ^{1}H NMR spectrum may be due to the hypervalent silicate ion, formed as a result of the interaction between acetate anion and TMSCN, as shown in the following spectra.[1-6]

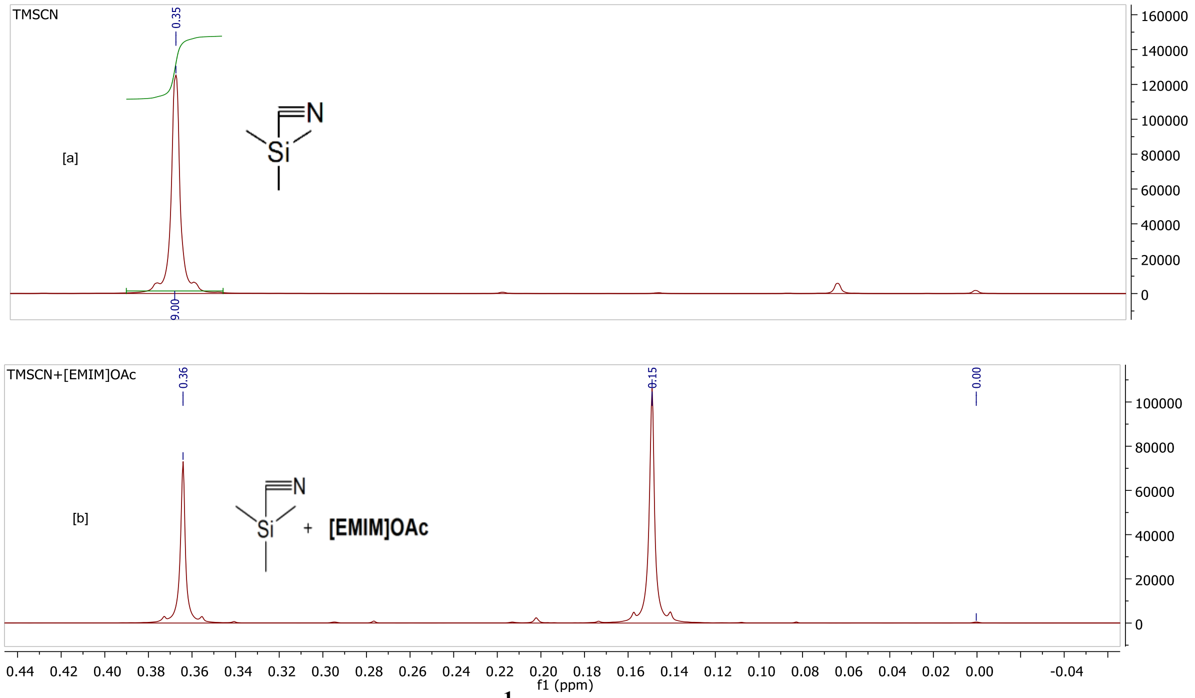

Supplementary Figure S1. Sections of ^{1}H NMR spectra of TMSCN in $CDCl_3$ solvent: [a] TMSCN only. [b] A 1:1 mixture of TMSCN and [EMIM]OAc (**1a**).

5. References.

1. George, S. C. & SUNG, S. K. Solvent-free cyanosilylation of carbonyl compounds catalyzed by NbCl5. *Bull. Korean Chem. Soc.* **28**, 1167-1170 (2007).
2. Liu, X., Qin, B., Zhou, X., He, B. & Feng, X. Catalytic asymmetric cyanosilylation of ketones by a chiral amino acid salt. *J. Am. Chem. Soc.* **127**, 12224-12225 (2005).
3. Kobayashi, S. & Nishio, K. Facile and highly stereoselective synthesis of homoallylic alcohols using organosilicon intermediates. *J Org Chem* **59**, 6620-6628 (1994).
4. Corriu, R., Dabosi, G. & Martineau, M. Stereochemical proof of the existence of nucleophilic activation at a silicon atom in nucleophilic substitution reactions. Substitution of the Si ・ Cl-bond with retention of configuration. *J. Organomet. Chem.* **154**, 33-43 (1978).
5. Holmes, I. P. & Kagan, H. B. The asymmetric addition of trimethylsilylcyanide to aldehydes catalysed by anionic chiral nucleophiles. Part 1. *Tetrahedron Lett.* **41**, 7453-7456 (2000).
6. Holmes, I. P. & Kagan, H. B. The asymmetric addition of trimethylsilylcyanide to aldehydes catalysed by anionic chiral nucleophiles. Part 2. *Tetrahedron Lett.* **41**, 7457-7460 (2000).
